# Flow carbonylation of sterically hindered *ortho*-substituted iodoarenes

**DOI:** 10.3762/bjoc.12.147

**Published:** 2016-07-19

**Authors:** Carl J Mallia, Gary C Walter, Ian R Baxendale

**Affiliations:** 1 Department of Chemistry, Durham University, South Road, Durham, DH1 3LE, United Kingdom; 2 Syngenta CP R&D Chemistry, Jealott's Hill International Research Centre, Bracknell, Berkshire, RG42 6EY, United Kingdom

**Keywords:** carbon monoxide, carbonylation of *ortho*-substituted substrates, flow chemistry, gases in flow, “tube-in-tube”

## Abstract

The flow synthesis of *ortho*-substituted carboxylic acids, using carbon monoxide gas, has been studied for a number of substrates. The optimised conditions make use of a simple catalyst system compromising of triphenylphosphine as the ligand and palladium acetate as the pre-catalyst. Carbon monoxide was introduced via a reverse “tube-in-tube” flow reactor at elevated pressures to give yields of carboxylated products that are much higher than those obtained under normal batch conditions.

## Introduction

Carbonylation reactions have received a great deal of attention both in batch as well as in flow (using plug/annular flow reactors [1–5] or “tube-in-tube” reactors [6–10]) and generally produce the desired products in good yields [11–14]. This is not the case though for the carbonylation of *ortho*-substituted substrates which are much more challenging as highlighted by the limited literature precedence [15–17]. However, these products are of considerable industrial importance, especially the amide and ester derivatives, which are commonly found in agrochemical active ingredients, for example tecloftalam, flutolanil, fluopyram and diflufenican. Likewise, in pharmaceutical compounds such as 2,4,5-trifluorobenzoic acid, which serve as a starting material for several antibacterial drugs such as ciprofloxacin (Cipro™), norfloxacin (Noroxin™) and pefloxacin (Peflacine™).

**Figure 1:**
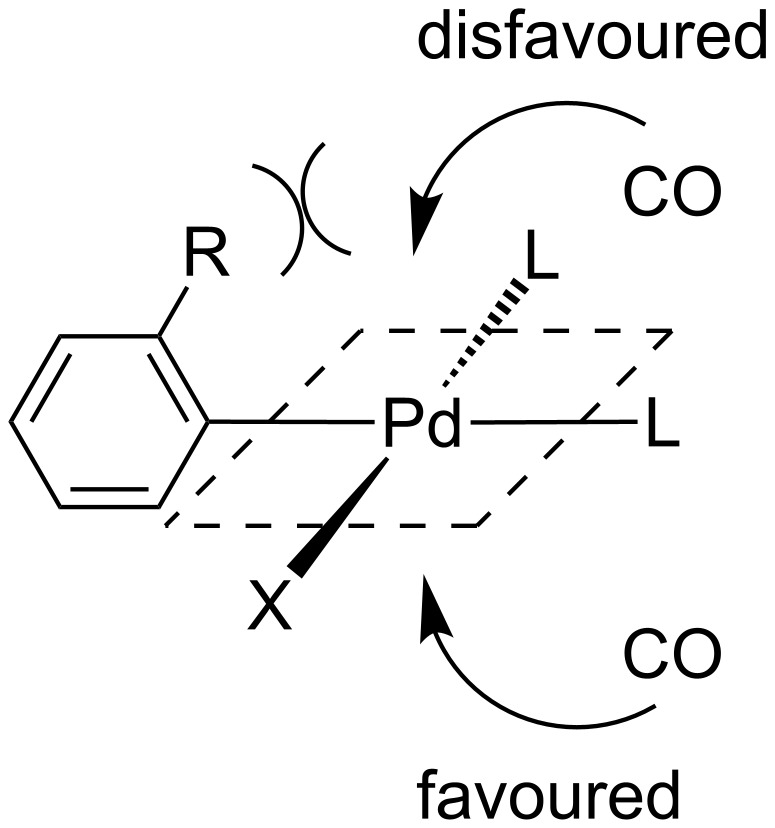
Steric interactions of the carbon monoxide coordination to the aryl complex intermediate.

**Figure 2:**
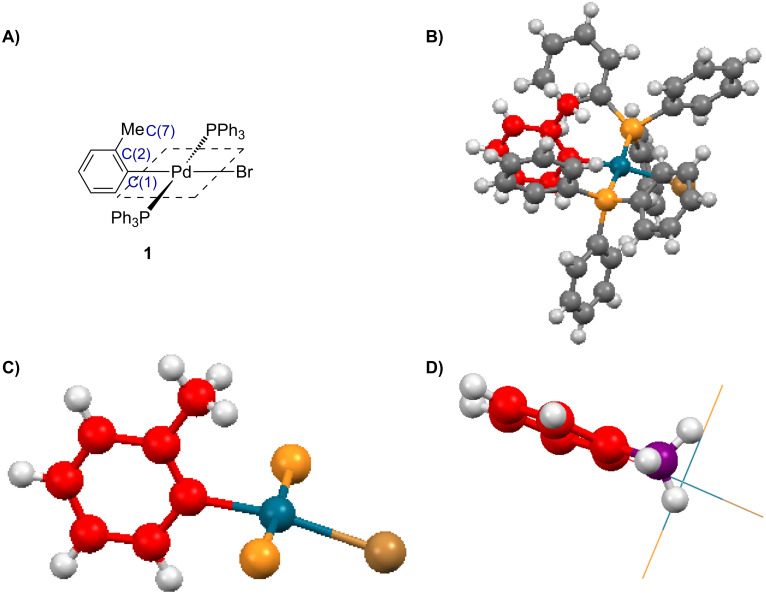
A) molecular structure of complex **1**; B) ball and stick representation of X-ray structure; C) ball and stick representation of X-ray structure showing the tolyl group only; D) topside view of X-ray structure [18].

The low catalyst turnover frequency (T.O.F.) and poor yields associated with *ortho*-substituted transformations are attributed to the carbon monoxide coordination to the intermediate aryl transition metal (i.e., Pd) complex which is inhibited by sterics [15]. Following oxidative addition of the aryl halide, an associative mechanism for the complexation of carbon monoxide on the d^8^ square planar intermediate would occur prior to the key migratory insertion step. In the complex, the aryl group would be oriented perpendicularly to the plane to minimise steric interactions thus placing the *ortho*-substituent directly over an axial site (Figure 1). The *ortho*-substituent therefore acts as a steric buttress hindering the approach of the incoming carbon monoxide thus slowing down the rate of the reaction. An X-ray structure of *trans*-bromo(*o*-tolyl)bis(triphenylphosphine)palladium(II) complex was reported by Cross et al. (Figure 2) [18]. The molecular structure of **1** comprises of a palladium atom with near perfect square planar geometry with a slight out of plane displacement of Br and C(1) where the Br–Pd–C(1) angle is 170.9°. As a whole, the molecule has approximate *C_s_* symmetry with the PPh_3_ ligands almost eclipsing each other if viewed along the P–Pd–P axis, with the tolyl group sandwiched between the two phenyl groups (Figure 2, structure B). Focusing on the tolyl group only, structure C (Figure 2) shows how the methyl of the tolyl group is placed straight over the axial position of the palladium. Structure D (Figure 2) is a top view of the crystal structure illustrating how the methyl group sits directly over the axial position of the palladium which would introduce steric effects inhibiting the CO coordination on the intermediate aryl complex.

As the carbonylation step becomes slower, the competing dehalogenation pathway becomes dominant resulting in overall lower yields of the carbonylated product. In principle, increasing the carbon monoxide concentration (by increasing the carbon monoxide pressure) together with an increase in temperature, should promote the carbonylation. However, an increase in carbon monoxide concentration can also decrease the amount of active Pd^0^ catalyst due to the π-acidic nature of carbon monoxide as a ligand, thus slowing down the reaction. Additionally, increasing the temperature will also increase the rate of side product formation. Consequently, optimisation of the carbon monoxide concentration and temperature is critical to obtaining a good yield of carbonylated *ortho*-substituted products.

## Results and Discussion

The application of flow chemistry [19–20] has been shown to be beneficial for many reactions that involve gases [21–29]. The efficient mixing along with high heat and mass transfer that are achieved through the use of small dimensioned channels such as those found in flow reactors, allow for the use of a wider range of reaction conditions which are otherwise difficult or impossible to achieve. The interfacial mixing area is also an important characteristic when gases are involved as this is an essential factor determining the solubility of a gas in the liquid phase. The interfacial area is generally very small when traditional batch chemistry equipment is used such as round bottom flasks. This also becomes proportionally smaller when larger volume flasks are used as in scale up procedures making the mass transfer even less efficient. In contrast, high interfacial areas can be achieved in flow reactors especially microchannel reactors (*a* = 3400–18000 m^2^ m^−3^) [30], which increases the mass transfer and thus helps solubilise the gases in the liquid phase.

**Figure 3:**
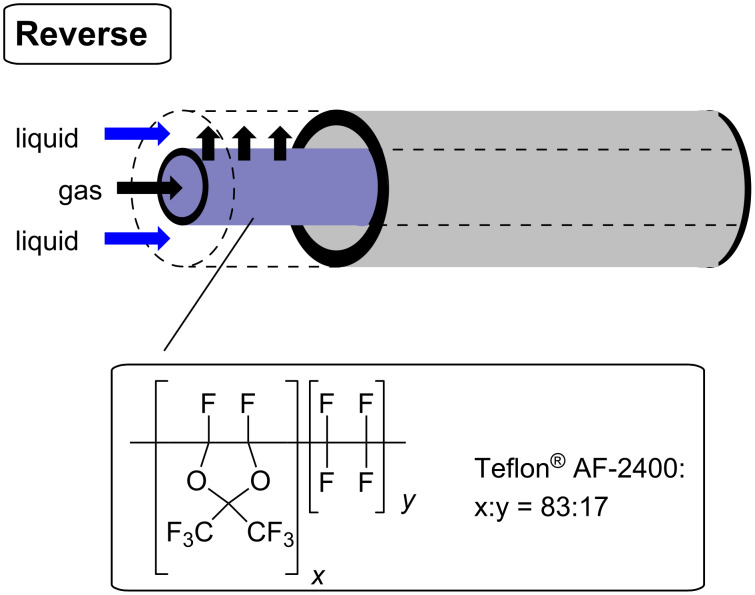
Reverse “tube-in-tube” reactor.

**Scheme 1:**
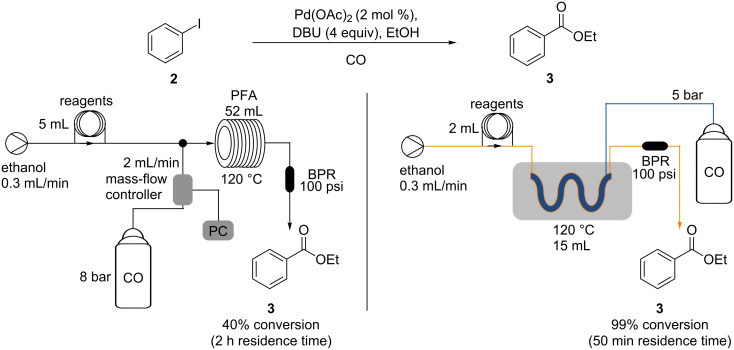
Comparison of plug flow reactor carbonylation (left) and “tube-in-tube” reactor carbonylation (right).

In our work a reverse “tube-in-tube” reactor [31–33] was used to deliver the carbon monoxide to the reaction (Figure 3), as this was shown to be more efficient than an alternative plug flow system (Scheme 1) when evaluated on iodobenzene (**2**).

**Scheme 2:**
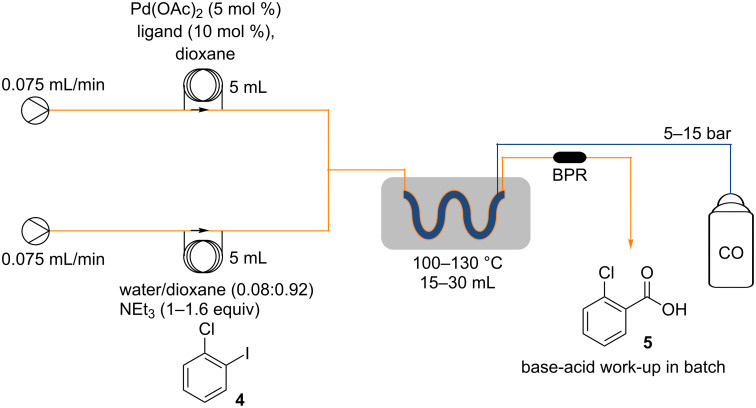
Schematic diagram of the flow process.

**Figure 4:**
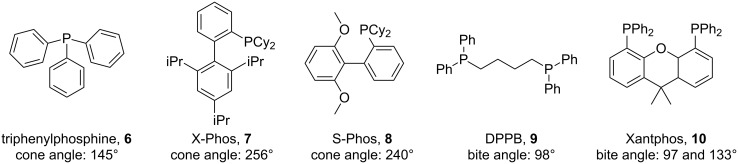
Phosphine ligands used for the *ortho*-carbonylation reaction.

The “tube-in-tube” gas-liquid unit was attached to a commercial flow system; Vapourtec R2+ Series along with an R4 heating unit. Having established the reactor design, we next used 2-chloro-1-iodobenzene (**4**) as a model substrate for screening and identification of a set of general reaction conditions (Scheme 2). Initially, a fixed 5 mol % of Pd(OAc)_2_ and 10 mol % of the phosphine ligand was investigated. It was noted that the catalyst level could be reduced [34], but this amount allowed for an efficient catalytic process with short reaction times in the region of two hours, a good match for the flow system assembly [8]. Five different phosphine ligands were subsequently tested, three of which were monodentate with a variable cone angle (**6**–**8**; 145–256°) [35–36] and the other two bidendate phosphine ligands namely 1,4-bis(diphenylphosphino)butane (DPPB, **9**; βn = 98°) and Xantphos (**10**; 104 and 133°) with differing bite angles (Figure 4) [37–39].

Initially using 5 bar of carbon monoxide and a temperature of 110 °C, the five ligands gave similar yields, with DPPB (**9**) giving marginally the highest and X-Phos (**7**) the lowest isolated yield. However, the highest selectivities for the desired product were obtained with S-Phos (**8**) and triphenylphosphine (**6**) (Table 1, entries 2 and 5), with the difference between the conversion and the isolated yield mainly equating to the dehalogenated product namely, chlorobenzene.

**Table 1:** Optimisation for the carbonylation of *ortho*-substituted substrates in flow.

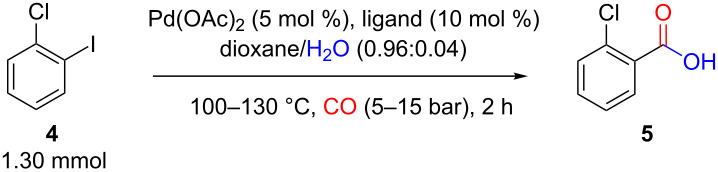					

Entry	Ligand	Temperature (°C)	CO pressure (bar)	Conversion (%)	Isolated yield of **5** (%)

1	X-Phos	110	5	68	31
2	S-Phos	110	5	43	36
3	DPPB	110	5	90	38
4	Xantphos	110	5	57	36
5	PPh_3_	110	5	44	36
6^a^	PPh_3_	110	5	59	36
7^b^	PPh_3_	110	5	80	33
8^c^	PPh_3_	110	5	N/D	18
9	PPh_3_	100	5	41	31
10	PPh_3_	120	5	60	37
11	PPh_3_	130	5	N/D	33
12^d^	PPh_3_	110	10	67	46
13^d^	PPh_3_	110	15	74	62
14^d,e^	PPh_3_	110	15	N/D	31
15^f^	PPh_3_	110	15	N/D	68
16^f,a^	PPh_3_	110	15	99	90
17^f,b^	PPh_3_	110	15	99	73

^a^1.6 equiv of base. ^b^2.0 equiv of base. ^c^1.1 equiv of DBU used instead of NEt_3_. ^d^10 mL reactor was not “tube-in-tube”. ^e^20 mol % DMF added. ^f^2 × 15 mL “tube-in-tube” reactors used. N/D: not determined.

Next changing the amount of triethylamine used from 1.1 equiv to 1.6 equiv and 2.0 equiv, respectively, did not significantly change the isolated yield of **5**. However, changing to the stronger base DBU (p*K*_a_ in water at 25 °C = 13.5) [40] dramatically reduced the isolated yield (Table 1, entry 8). A wider temperature range was also investigated (Table 1, entries 9–11). This resulted in only a small increase in the yield on going from 100 °C to 120 °C and a marginal decrease when the temperature was further increased to 130 °C. As there was no significant difference between 110 °C and 120 °C (Table 1, entries 5 and 10), the lower temperature was selected for the use in the next set of experiments. Interestingly the addition of up to 20 mol % of dimethylformamide (DMF) as an additive did not improve the yield which had been suggested by evaluation of similar reactions in the literature [6,10]. However as anticipated, an increase in carbon monoxide pressure did pertain to a raise in product yield to 62% (Table 1, entries 12 and 13). In addition the effect of gas contact time was evaluated by employing two “tube-in-tube” reactors linked in series; albeit this resulted in only a modest improvement in yield (Table 1, entry 15). A further increase in product yield was observed when a larger excess of the triethylamine base (1.6 equiv) was used (Table 1, entry 16), but the isolated yield dropped with further equivalents of triethylamine (2.0 equiv; Table 1, entry 17). This indicated that the reaction was being inhibited by low pH which was generated at higher conversions when insufficient base was present to neutralise the carboxylic acid being formed. Interestingly, the requirement for a higher excess of base during initial screening (Table 1, entries 6 and 7) had been masked due to the initial low conversions achieved.

**Scheme 3:**
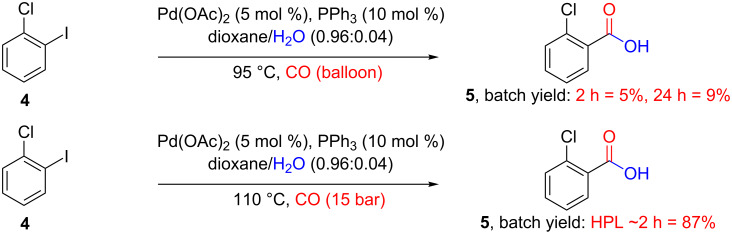
The batch carbonylation of 2-chloro-1-iodobenzene in conventional lab (top) and using a Parr autoclave in high pressure lab (bottom).

For comparison purposes, two batch carbonylation reactions were performed. The first of these batch reactions (conducted in a conventional laboratory set-up) used the palladium triphenylphosphine catalyst system under refluxing conditions with a double-walled balloon to deliver the carbon monoxide (Scheme 3). This would constitute a normal set-up used by many laboratory chemists when reactions involving gases are attempted if no specialised equipment is available. Two parallel reactions were preformed, one reaction was quenched after 2 hours and after purification yielded 5% of product **5**, while the second reaction was quenched after 24 h yielding 9% of purified **5**. The difference in the yields obtained in batch when compared to the reactions conducted in flow, most probably arises from the fact that not enough carbon monoxide is being delivered to the reaction mixture. The dehalogenation pathway is then preferred yielding chlorobenzene as the main product.

The second batch reaction set-up, conducted in the departmental high pressure lab (HPL), was set up in a Parr autoclave using carbon monoxide at 15 bar and 110 °C for 2 hours. After purification, a yield of 87% for product **5** was obtained. This compares well with the flow protocol, however, the reaction “processing” time is in reality much longer due to the long cooling and heating times (4 h 15 min “processing” time, see experimental section in Supporting Information File 1 for more details). Also, the time required due to the extra precautionary measures needed when high pressure laboratory equipment is used means that the turnaround time is much longer. This makes the flow reactor more efficient in terms of processing time. Additionally, the added safety and potential benefits regarding scale up associated with the flow reactor makes this even more favourable.

**Scheme 4:**
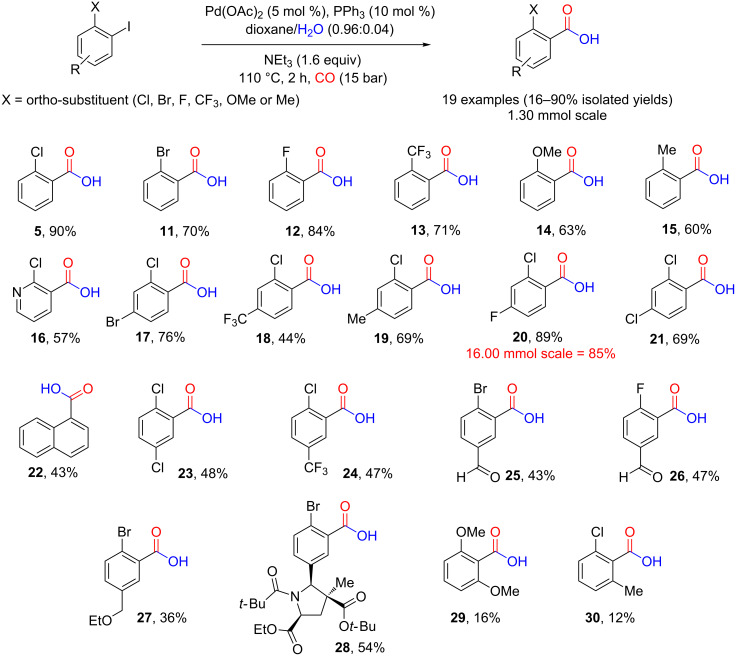
Structures of *ortho*-substituted carboxylic acids prepared via a continuous flow hydroxy-carbonylation method.

Having identified a set of reaction conditions for successful carbonylation, a number of additional substrates were assessed to determine the generality of the flow process. No significant impact was seen on the overall yield by altering the *ortho*-substituent to a bromo, fluoro or trifluoromethyl group. However, a slight decrease associated with the larger sizes of bromo and trifluoromethyl groups may be inferred (Scheme 4, **11**, **13**). A more pronounced decrease in yield was obtained for substrates **14** and **15** (Scheme 4, 63% and 60%, respectively) probably due to the larger size of these groups and as well as electronic effects (the more electron withdrawing trifluoromethyl group substrate **13** gave a 71% yield). For comparisons of the sizes of the *ortho*-substituents used, A-values can be used as a guide (Cl: 0.43 kcal/mol, Br: 0.38 kcal/mol, F: 0.15 kcal/mol, OMe: 0.60 kcal/mol, CF_3_: 2.10 kcal/mol and Me: 1.70 kcal/mol) [41]. This indicates interplay between electronic and steric factors.

**Scheme 5:**
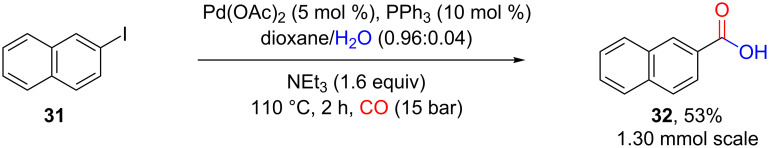
Flow carbonylation of 2-iodonaphtalene.

Using a pyridine as a heteroaromatic substrate gave a lower but still acceptable yield of **16** compared to the phenyl equivalent (**5**). In general, substitution at the 4-position of the aryl gave moderate to good yields (Scheme 4, **17**–**21**) with weakly electron-withdrawing substituents or electron-donating groups giving better yields (Scheme 4, compounds **17**, **19**–**21**) than the more electron-withdrawing CF_3_ group (Scheme 4, compound **18**). In the case of **22** the attached aromatic ring introduces both the ortho substituted sterics and the electronic effects from the additional aromatic ring attached. For comparison 2-iodonaphthalene (**31**) was carboxylated under the same conditions to give 2-naphthoic acid (**32**) showing that reducing the steric encumbrance at the ortho position improves the yield by 10% for this substrate (Scheme 5).

**Figure 5:**
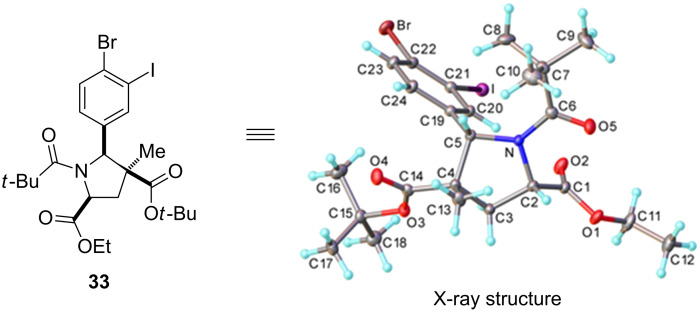
X-ray structure of substrate **33**.

Moderate yields were obtained with 5-substited substrates (Scheme 4, compounds **23**–**30**). Both electron-withdrawing groups (Scheme 4, compounds **23**–**26**) and electron-donating groups gave similar yields (Scheme 4, compounds **27** and **28**) indicating that the inductive effects are not affecting the yields. Comparing the yields obtained for **27** and **28** also indicates that the sterics at the 5-position are not affecting the yield with a large group at the 5-position of substrate **30** [42] (see X-ray structure of substrate **33**, Figure 5) actually leading to a better yield than obtained for product **27** which contains the smaller ethoxy group at the 5-position.

The lowest yields of the array were obtained for compounds **29** and **30**, demonstrating the importance of sterics and electronics adjacent to the leaving group. In both cases, the carbon monoxide insertion is assumed to be slow as both axial positions of the aryl complex would be hindered, meaning the competing proton-dehalogenation pathway becomes preferred, giving 1,3-dimethoxybenzene as the main product, which was isolated in 31% yield in the case of **29** and 3-chlorotoluene in the case of **30** which was isolated in 52% yield (Scheme 4).

**Scheme 6:**
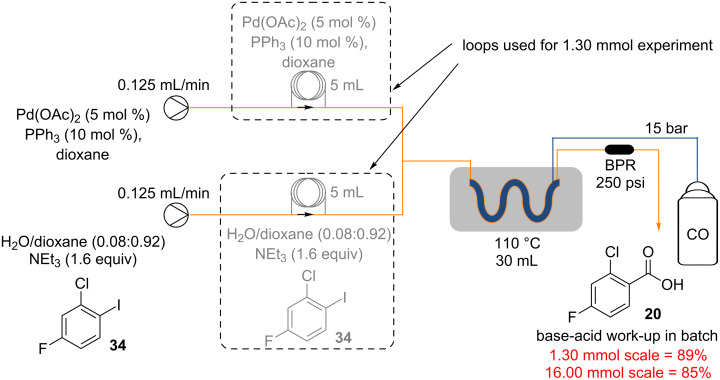
Scale up synthesis of 2-chloro-4-fluorobenzoic acid (**20**).

To demonstrate the potential scalability of the reaction conditions, the synthesis of compound **20** was repeated at 16 mmol scale, a factor of twelve times the original 1.3 mmol test scale (Scheme 6). The yield obtained for the larger scale was 85% which is consistent with the original 89% obtained at the 1.30 mmol scale, indicating that the processes is robust and reliably delivering 1.19 g h^−1^ of **20** in 85% isolated yield.

## Conclusion

We have successfully demonstrated how flow chemistry can be used to enhance difficult transformations such as the palladium-catalysed hydroxy-carbonylation of *ortho*-substituted iodoarenes. The optimised conditions were also demonstrated to work on a number of *ortho*-substituted substrates giving moderate to good yields. Comparison of **22** with **32** also showed that the steric encumbrance on the ortho position has an effect on the yield even when other electronic effects are in place such as those coming from the additional aromatic ring attached. A scale-up of the reaction conditions was performed providing comparable yields to those obtained from the initial smaller test scale. This method could thus be an efficient and scalable approach to synthesising important intermediates containing *ortho*-substituted carboxylic acids.

## Experimental

See Supporting Information File 1 for full experimental data.

### General notes

#### Warning

Carbon monoxide is highly toxic and extremely flammable gas. All reactions were carried out in well ventilated fume cupboards and carbon monoxide detectors were continuously used thought the process. High pressure lab facilities were used under the supervision of dedicated staff and all associated safety measures were taken. Parr autoclave was pressure tested at 80 bar before use.

## Supporting Information

File 1 Experimental part.

File 2 X-ray information data of compound **33**.
