# Supplementary material for: Flow carbonylation of sterically hindered ortho-substituted iodoarenes
Source: Beilstein J Org Chem. 2016 Jul 19;12:1503–11. doi: 10.3762/bjoc.12.147 (PMC4979912; doi:10.3762/bjoc.12.147)
Supplement: File 1 — Experimental part. [file Beilstein_J_Org_Chem-12-1503-s001.pdf]

# Supporting Information

for

# Flow carbonylation of sterically hindered *ortho*-substituted iodoarenes

Carl J. Mallia,[1] Gary C. Walter[2] and Ian R. Baxendale*[1]

Address: [1]Department of Chemistry, Durham University, South Road, Durham, DH1 3LE, United Kingdom and [2]Syngenta CP R&D Chemistry, Jealott's Hill International Research Centre, Bracknell, Berkshire, RG42 6EY, United Kingdom

Email: Ian R. Baxendale - i.r.baxendale@durham.ac.uk

*Corresponding author

## Experimental part

Unless specified, reagents were obtained from commercial sources and used without further purification. Solvents were obtained from Fisher scientific, and $H_2O$ was deionised before use.
NMR spectra were recorded on either Bruker Avance-400, Varian VNMRS-600 or Varian VNMRS-700 instrument and was calibrated to the residual solvent according to the literature.[1] Assignments are based on DEPT-135, COSY, NOESY, HSQC and HMBC spectra.

Liquid chromatography-mass spectrometry (LCMS) was performed on an Agilent HP 1100 series chromatograph (Mercury Luna 3μ C18 (2) column) attached to a Waters ZQ2000 mass spectrometer with ESCi ionisation source in ESI mode. Elution was carried out at a flow rate of 0.6 mL/min using a reverse phase gradient of MeCN–water containing 0.1% formic acid. Gradient = 0–1 min: hold MeCN 5%, 1–4 min: ramp MeCN 5–95%, 4–5 min: hold MeCN 95%, 5–7 min: ramp MeCN 95–5%, 7–8 min: hold MeCN 5%. Retention times are reported as Rt. High resolution mass spectra (HRMS) were recorded on a Waters Micromass LCT Premier spectrometer using time of flight with positive electrospray ionisation (ESI+), an ABI/MDS Sciex Q-STAR Pulsar with ESI+ and an ASAP (atmospheric pressure solids analysis probe ionisation), or a Bruker BioApex II 4.7e FTICR utilising either ESI+ or a positive electron ionisation (EI+) source equipped with a direct insertion probe. The mass reported is that containing the most abundant isotopes ($^{35}$Cl and $^{79}$Br). Limit: ± 5 ppm.

IR spectra were recorded neat on a Perkin-Elmer Spectrum Two FT-IR spectrometer using Universal ATR sampling accessories. Letters in parentheses refer to the relative absorbency of the peak: w – weak (<40% of the most intense peak), m – medium (40–75% of the most intense peak), s – strong (>75% of the most intense peak) and br – broad.

Melting points were recorded on an Optimelt automated melting point system with a heating rate of 1 °C/min (70% onset point and 10° clear point) and are uncorrected.

X-ray diffraction experiment was carried out on a D8 Venture 3-circle Bruker AXS diffractometer with a PHOTON 100 CMOS area detector, using graphite-monochromated Mo $K_\alpha$ radiation ($\bar{\lambda}$ = 0.71073 Å) from IμS microsource and a Cryostream (Oxford Cryosystems) open-flow $N_2$ cryostat. The structure was solved by direct methods (SHELXS 2013/1 software[2]) and refined by full-matrix least squares against $F^2$ of all reflections, using OLEX2[3] and SHELXL 2014/7 software.[4] Crystallographic data for structure **29** have been deposited with the Cambridge Crystallographic Data Centre as supplementary publication CCDC-1470506.

**General procedures:**

***Ortho-substituted carbonylation in flow using a "tube-in-tube" reactor.***

For a typical reaction, a Vapourtec 2R+ Series was used as the platform with a Vapourtec Gas/Liquid Membrane Reactor to load the carbon monoxide. The HPLC pump were both set at 0.125 mL/min, temperature of the reactor at 110 °C, pressure of CO at 15 bar with a back pressure regulator of 250 psi (17.24 bar). The system was left running for 2 h to reach steady state after which time the flow streams were switched to pass from the loops where the substrates and catalysts were loaded. The first loop (5 mL) was filled with a solution of palladium acetate (20 mg, 0.08 mmol), triphenylphosphine (48 mg, 0.168 mmol) in 6 mL of 1,4-dioxane while the second loop (5 mL) was filled with a solution made from the ortho-substituted iodoarene substrate (1.68 mmol), triethylamine (0.272 g, 0.374 mL, 2.69 mmol) and water (0.505 g, 28 mmol) in 5.8 mL of 1,4-dioxane.

An Omnifit® column filled with 1.71 $cm^3$ (r = 0.33 cm, h = 5.00 cm) of cotton was positioned just before the back pressure regulator to trap any particulate matter formed to avoid blocking of the back pressure regulator. After the substrates were passed through the system, the outlet of the flow stream was directed into a receptacle where the excess carbon monoxide gas was vented off in the fume cupboard.  The reaction mixture was then evaporated to dryness, ethyl acetate (25 mL) and sodium carbonate solution (2 M, 10 mL) were added and transferred to a separating funnel. After collecting the aqueous layer, the organic layer was extracted with sodium carbonate solution (2 M, 2 × 10 mL). The combined aqueous layers were acidified by the addition of 2 M HCl solution which was then extracted with ethyl acetate (3 x 25 mL). The organic layer was dried over sodium sulfate, and the solvent evaporated under vacuum to give the crude product as a solid. The crude product was then recrystallised from the appropriate solvent.

***Heck-carbonylation in flow using the plug flow reactor.***

For the Heck-carbonylation of iodobenzene and ethanol, a mixture of 1.64 mL DBU (11.0 mmol) and 5 mL of ethanol was made to which palladium acetate (5 mg, 0.022 mmol) was added and left stirring for 15 minutes to obtain a pale orange solution. Iodo benzene (2.05 g, 10.0 mmol) was then added to this solution and left stirring for 5 minutes. This solution was then injected in the flow reactor (Uniqsis FlowSyn), at the following conditions: coil temperature of 120 °C, carbon monoxide rate of 2 mL/min at 8 bar of pressure (supplied using EL-Flow Select Bronkhorst flow meter), solvent flow rate of 0.3 mL/min, 100 psi BPR

and a residence time of 2 hours. The crude was purified on a silica column (9:1, hexane: ethyl acetate) to obtain 0.6 g (41% isolated yield) as a pale yellow oil.

*Note:* The flow reactor was equilibrated at the appropriate conditions for 2 h prior to the addition of the substrates. Additionally, by using a flow of 2 mL/min of carbon monoxide it was ensured that equal size plugs (~0.5 cm long) of gas/liquid were obtained allowing for large interfacial areas, which should ensure carbon monoxide saturation in the liquid plug over the 2 h residence time.

***Ortho-substituted carbonylation in batch (Conventional Lab).***

A solution of palladium acetate (20 mg, 0.08 mmol) and triphenylphosphine (48 mg, 0.168 mmol) in 11.8 mL of 1,4-dioxane was prepared, to which 2-chloro-iodobenzene (0.401g, 1.68 mmol), triethylamine (0.272 g, 0.374 mL, 2.69 mmol) and water (0.505 g, 28 mmol) were added in a $N_2$ filled 25 mL flask. A balloon (made from two balloons inside each other) of carbon monoxide was attached to the flask and the flask was emptied through an empty needed. This process was repeated once and then the third time the carbon monoxide in the balloon was not emptied by removing the empty needle. The reaction was heated to reflux, cooled after 2 h or 24 h, solvent evaporated and the same extraction/purification for the flow protocol was repeated using acetonitrile to recrystallise the product.

***Ortho-substituted carbonylation in batch (High-Pressure Lab).***

A solution of palladium acetate (80 mg, 0.32 mmol) and triphenylphosphine (192 mg, 0.672 mmol) in 24 mL of 1,4-dioxane was prepared and stirred for 15 min, to which a solution of 2-chloro-iodobenzene (1.602 g, 6.72 mmol), triethylamine (1.088 g, 1.50 mL, 10.76 mmol) and water (2.02 g, 112 mmol) in 24 mL of 1,4-dioxane was added in the Parr autoclave (stainless steel, 100 mL capacity, 200 bar maximum pressure). The autoclave was tightly sealed and placed in the heating rig. The autoclave was then purged with nitrogen (3 × 10 bar) and with carbon monoxide (3 × 10 bar), keeping 10 bar of carbon monoxide in the autoclave at which point it was heated to 110 °C over 15 minutes. The carbon monoxide pressure was adjusted to 15 bar and the reaction was left stirring for 2 h and was then cooled down to 30 °C over 2 h. The autoclave was then purged with nitrogen (4 × 10 bar) and the reaction mixture removed from the autoclave. The same extraction/purification sequence as for the flow protocol was repeated.

**Spectroscopic data for products:**

**2-Chlorobenzoic acid, [CAS Number: 118-91-2], 4:**

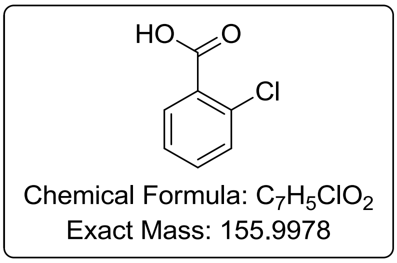


Isolated yield: 0.183 g (90%, 1.30 mmol scale), pale yellow crystals (recrystallised from MeCN); $^1$H NMR (400 MHz, DMSO-*d*$_6$) δ/ppm 13.42 (s, br, 1H), 7.78 (d, *J* = 7.6 Hz, 1H), 7.68 – 7.17 (m, 3H); $^{13}$C NMR (151 MHz, DMSO- *d*$_6$) δ/ppm 167.2 (C), 133.0 (CH), 132.0 (C), 131.9 (C), 131.2 (CH), 131.1 (CH), 127.7 (CH); IR (neat) ν = 1683 (s, C=O) cm$^{-1}$; LC-MS (MeCN), Rt. 2.34 min, m/z = 157.0 $[M+H]^+$. HR-MS (ESI-TOF) calculated for $C_7H_6O_2Cl$ 157.0056, found 157.0063 (Δ = 4.5 ppm). M.p. 140-141 °C (MeCN) (Literature: 139-140 °C).[5]

**2-Bromobenzoic acid, [CAS Number: 88-65-3], 10:**

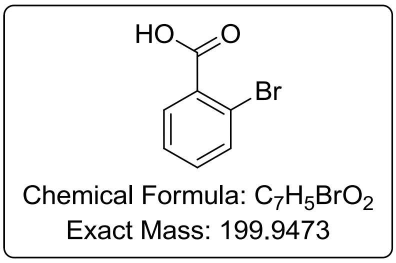


Isolated yield: 0.185 g (70%, 1.30 mmol scale), white crystals (recrystallised from MeCN); $^1$H NMR (400 MHz, DMSO-*d*$_6$) δ/ppm 13.39 (s, br, 1H), 7.78 – 7.66 (m, 2H), 7.50 – 7.40 (m, 2H); $^{13}$C NMR (151 MHz, DMSO- *d*$_6$) δ/ppm 167.4 (C), 133.7 (CH), 132.5 (CH), 130.6 (CH), 127.7 (CH), 127.5 (C), 119.9 (C); IR (neat) ν = 1675 (s, C=O) cm$^{-1}$; LC-MS (MeCN), Rt. 2.27 min, m/z = 200.8 $[M+H]^+$. HR-MS ($^+$ESI-TOF) calculated for $C_7H_6O_2Br$ 200.9551, found 200.9554 (Δ = 1.49 ppm). M.p. 147-149 °C (MeCN) (Literature: 147-149 °C).[5]

**2-Fluorobenzoic acid, [CAS Number: 445-29-4], 11:**

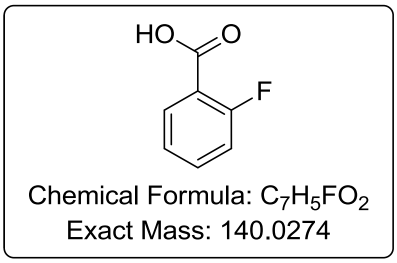


Isolated yield: 0.152 g (84%, 1.30 mmol scale), yellow crystals (recrystallised from MeCN); $^1$H NMR (400 MHz, DMSO-*d*$_6$) δ/ppm 13.23 (s, br, 1H), 7.87 (td, *J* = 7.6, 1.7 Hz, 1H), 7.64 (dddd, *J* = 7.9, 6.9, 5.0, 1.9 Hz, 1H), 7.35 – 7.26 (m, 2H); $^{13}$C NMR (151 MHz, DMSO- *d*$_6$) δ/ppm 165.5 (d, *J* = 3.0 Hz, C), 161.5 (d, *J* = 256.7 Hz, C), 135.1 (d, *J* = 8.9 Hz, CH), 132.3

(CH), 124.9 (d, $J$ = 3.8 Hz, CH), 119.8 (d, $J$ = 10.3 Hz, C), 117.4 (d, $J$ = 22.1 Hz, CH); $^{19}$F NMR (376 MHz, DMSO-$d_6$) δ/ppm -110.65 (s); IR (neat) ν = 1685 (s, C=O) cm$^{-1}$; LC-MS (MeCN), Rt. 2.06 min, m/z = 139.1 [M-H]$^-$. HR-MS ($^-$ESI-TOF) calculated for $C_7H_4O_2F$ 139.0195, found 139.0189 (Δ = 4.3 ppm). M.p. 122-124 °C (MeCN) (Literature: 123-125 °C).[6]

**2-(Trifluoromethyl)benzoic acid, [CAS Number: 433-97-6], 12:**

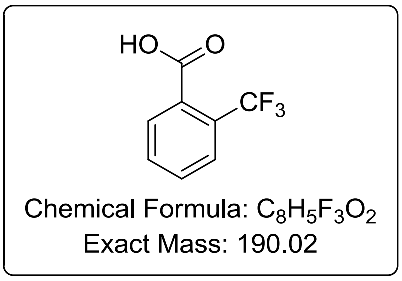


Isolated yield: 0. 0.176g (71%, 1.30 mmol scale), white crystals (recrystallised from MeCN); $^{1}$H NMR (400 MHz, $CDCL_3$) δ/ppm 11.23 (s, br, 1H), 8.05 – 7.97 (m, 1H), 7.86 – 7.78 (m, 1H), 7.73 – 7.63 (m, 2H); $^{13}$C NMR (151 MHz, $CDCl_3$) δ/ppm 172.2 (C), 132.4 (CH), 132.0 (CH), 131.3 (CH), 129.7 (q, $J$ = 2.7 Hz, C), 129.7 (q, $J$ = 49.2 Hz, C), 127.2 (q, $J$ = 8.5 Hz, CH), 123.3 (q, $J$ = 410.3 Hz, C); $^{19}$F NMR (376 MHz, DMSO-$d_6$) δ/ppm -59.32 (s); IR (neat) ν = 1700 (s, C=O) cm$^{-1}$; LC-MS (MeCN), Rt. 2.47 min, m/z = 189.4 [M-H]$^-$. HR-MS ($^-$ESI-TOF) calculated for $C_8H_5F_3O_2$ 189.0163, found 189.0171 (Δ = 4.2 ppm). M.p. 108-109 °C (MeCN) (Literature: 108-110 °C).[7]

**2-Methoxybenzoic acid, [CAS Number: 579-75-9], 13:**

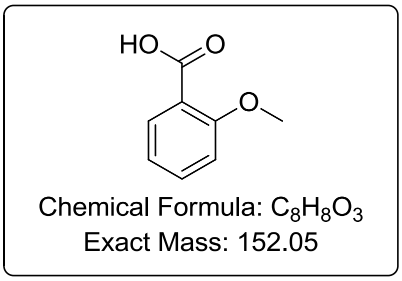


Isolated yield: 0.125 g (63%, 1.30 mmol scale), white crystals (recrystallised from MeCN); $^{1}$H NMR (400 MHz,$CDCl_3$) δ/ppm 10.76 (s, br, 1H), 8.18 (dd, $J$ = 7.8, 1.8 Hz, 1H), 7.58 (ddd, $J$ = 8.3, 7.3, 1.8 Hz, 1H), 7.14 (td, $J$ = 7.6, 1.0 Hz, 1H), 7.06 (dd, $J$ = 8.4, 1.1 Hz, 1H), 4.08 (s, 3H); $^{13}$C NMR (151 MHz, $CDCl_3$) δ/ppm 165.6 (C), 158.2 (C), 135.2 (CH), 133.9 (CH), 122.3 (CH), 117.7 (C), 111.7 (CH), 56.8 ($CH_3$); IR (neat) ν = 1668 (s, C=O) cm$^{-1}$; LC-MS (MeCN), Rt. 2.03 min, m/z = 153.3 [M+H]$^+$. HR-MS ($^+$ESI-TOF) calculated for $C_8H_9O_3$ 153.0552, found 153.0554 (Δ = 1.3 ppm). M.p. 101-102 °C (MeCN) (Literature: 100-102 °C).[8]

**2-Methylbenzoic acid, [CAS Number: 118-90-1], 14:**

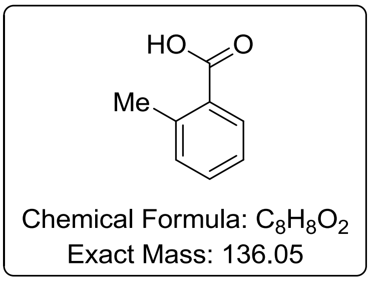


Isolated yield: 0.106 g (60%, 1.30 mmol scale), white crystals (recrystallised from MeCN); $^{1}$H NMR (400 MHz, $CDCl_3$) δ/ppm 11.65 (s, br, 1H), 8.13 – 8.05 (m, 1H), 7.47 (td, *J* = 7.5, 1.5 Hz, 1H), 7.30 (t, *J* = 7.4 Hz, 2H), 2.68 (s, 3H); $^{13}$C NMR (151 MHz, $CDCl_3$) δ/ppm 173.6 (C). 141.5 (C), 133.1 (CH), 132.1 (CH), 131.7 (CH), 128.4 (C), 126.0 (CH), 22.3 ($CH_3$); IR (neat) ν = 1679 (s, C=O) $cm^{-1}$; LC-MS (MeCN), Rt. 2.58 min, m/z = 135.4 $[M-H]^{-}$. HR-MS ($^{-}$ESI-TOF) calculated for $C_8H_7O_3$ 135.0446, found 135.0448 (Δ = 1.5 ppm). M.p. 103-104 °C (MeCN) (Literature: 104-105 °C).[8]

**2-chloronicotinic acid, [CAS Number: 2942-59-8], 15:**

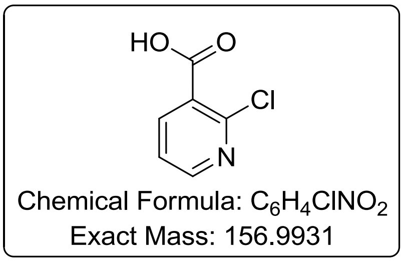


Isolated yield: 0.116 g (57%, 1.30 mmol scale), yellow crystals (recrystallised from EtOH); $^{1}$H NMR (400 MHz, DMSO-$d_6$) δ/ppm 13.78 (s, br, 1H), 8.56 (dd, *J* = 4.8, 2.0 Hz, 1H), 8.23 (dd, *J* = 7.7, 2.0 Hz, 1H), 7.54 (dd, *J* = 7.7, 4.8 Hz, 1H); $^{13}$C NMR (151 MHz, DMSO- $d_6$) δ/ppm 165.8 (C), 151.7 (CH), 147.8 (C), 140.0 (CH), 128.2 (C), 123.2 (CH); IR (neat) ν = 1701 (s, C=O) $cm^{-1}$; LC-MS (MeCN), Rt. 1.79 min, m/z = 156.0 $[M-H]^{-}$. HR-MS ($^{-}$ESI-TOF) calculated for $C_6H_3NO_2Cl$ 155.9852, found 155.9848 (Δ = 2.6 ppm). M.p. 183 °C (EtOH, decomposed) (Literature: 190-192 °C).[9]

**4-bromo-2-chlorobenzoic acid, [CAS Number: 59748-90-2], 16:**

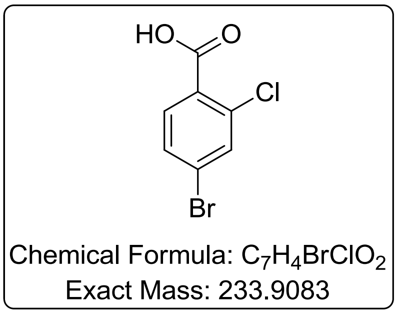


Isolated yield: 0.232 g (76%, 1.30 mmol scale), white crystals (recrystallised from MeCN); $^{1}$H NMR (400 MHz, DMSO-$d_6$) δ/ppm 13.55 (s, br, 1H) 7.86 (d, *J* = 1.9 Hz, 1H), 7.75 (d, *J* = 8.3 Hz, 1H), 7.66 (dd, *J* = 8.3, 1.9 Hz, 1H); $^{13}$C NMR (151 MHz, DMSO- $d_6$) δ/ppm 165.9 (C), 133.0 (C), 132.9 (CH), 132.5 (CH), 130.5 (C), 130.4 (CH), 125.1 (C); IR (neat) ν = 1676 (s, C=O) $cm^{-1}$; LC-MS (MeCN), Rt. 2.56 min, m/z = 232.9 $[M-H]^{-}$. HR-MS ($^{-}$ESI-TOF)

calculated for $C_7H_3O_2ClBr$ 232.9005, found 232.9013 (Δ = 3.43 ppm). M.p. 171-172°C (MeCN) (Literature: 170-172 °C).[10]

**2-chloro-4-(trifluoromethyl)benzoic acid, [CAS Number: 23228-45-7], 17:**

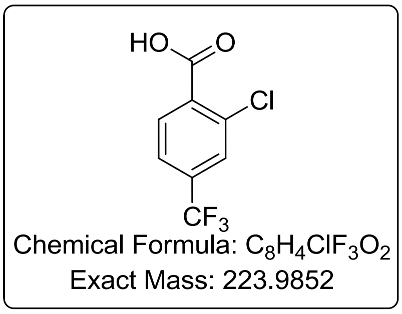


Isolated yield: 0.128 g (44%, 1.30 mmol scale), white crystals (recrystallised from MeCN); $^{1}$H NMR (400 MHz, DMSO-$d_6$) δ/ppm 13.85 (s, br, 1H), 8.01 – 7.94 (m, 2H), 7.82 (dd, *J* = 8.3, 1.7 Hz, 1H); $^{13}$C NMR (151 MHz, DMSO- $d_6$) δ/ppm 165.9 (s, C), 135.8 (s, CH), 132.16 (q, *J* = 32.3 Hz, C), 132.15 (s, C), 131.4 (s, C), 127.4 (q, *J* = 3.8 Hz, CH), 124.2 (q, *J* = 3.8, 3.0 Hz, CH), 121.6 (s, C); $^{19}$F NMR (376 MHz, DMSO-$d_6$) δ/ppm -61.62 (s); IR (neat) ν = 1685 (s, C=O) cm$^{-1}$; LC-MS (MeCN), Rt. 2.68 min, m/z = 223.0 $[M-H]^-$. HR-MS ($^-$ESI-TOF) calculated for $C_8H_3O_2F_3Cl$ 222.9774, found 222.9778 (Δ = 1.8 ppm). M.p. 114-115°C (MeCN) (Literature: 114-116 °C).[9]

**2-Chloro-4-methylbenzoic acid, [CAS Number: 7697-25-8], 18:**

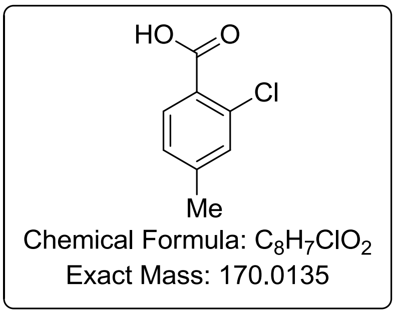


Isolated yield: 0.152 g (69%, 1.30 mmol scale), grey crystals (recrystallised from MeCN); $^{1}$H NMR (400 MHz, DMSO-$d_6$) δ/ppm 13.19 (s, br, 1H), 7.72 (d, *J* = 7.9 Hz, 1H), 7.38 (dd, *J* = 1.7, 0.9 Hz, 1H), 7.24 (ddd, *J* = 7.9, 1.7, 0.8 Hz, 1H), 2.34 (s, 3H); $^{13}$C NMR (151 MHz, DMSO- $d_6$) δ/ppm 166.5 (C), 143.3 (C), 131.9 (C), 131.1 (CH), 131.0 (CH), 128.1 (C), 127.8 (CH), 20.5 ($CH_3$); IR (neat) ν = 1675 (s, C=O) cm$^{-1}$; LC-MS (MeCN), Rt. 2.41 min, m/z = 171.3$[M+H]^+$. HR-MS ($^+$ESI-TOF) calculated for $C_8H_8O_2Cl$ 171.0213, found 171.0207 (Δ = 3.5 ppm). M.p. 155-156 °C (MeCN) (Literature: 150-152 °C).[11]

**2-Chloro-4-fluorobenzoic acid, [CAS Number: 2252-51-9], 19:**

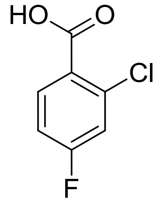


Chemical Formula: $C_7H_4ClFO_2$
Molecular Weight: 174.5569

Isolated yield: 0.154 g (89%, 1.30 mmol scale) or 2.372 g (85%, 16.00 mmol scale), white crystals (recrystallised from MeCN); $^1$H NMR (400 MHz, DMSO-$d_6$) δ/ppm 13.45 (s, br, 1H), 7.91 (dd, $J$ = 8.7, 6.3 Hz, 1H), 7.56 (dd, $J$ = 9.0, 2.6 Hz, 1H), 7.32 (ddd, $J$ = 8.8, 8.2, 2.6 Hz, 1H); $^{13}$C NMR (151 MHz, DMSO- $d_6$) δ/ppm 165.7 (s, C), 163.2 (d, $J$ = 252.6 Hz, C), 133.7 (d, $J$ = 11.1 Hz, C), 133.3 (d, $J$ = 9.8 Hz, CH), 127.7 (d, $J$ = 3.5 Hz, C), 118.1 (d, $J$ = 25.2 Hz, CH), 114.60 (d, $J$ = 21.4 Hz, CH); $^{19}$F NMR (376 MHz, DMSO-$d_6$) δ/ppm -106.76 (s); IR (neat) ν = 1670 (s, C=O) cm$^{-1}$; LC-MS (MeCN), Rt. 2.31 min, m/z = 173.1 [M-H]$^-$. HR-MS ($^-$ESI-TOF) calculated for $C_7H_3O_2FCl$ 172.9806, found 172.9810 (Δ = 2.3 ppm). M.p. 185-186 °C (MeCN) (Literature: 180-181 °C).[12]

**2,4-dichlorobenzoic acid, [CAS Number: 50-84-0], 20:**

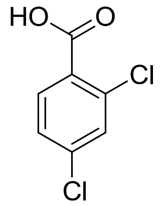


Chemical Formula: $C_7H_4Cl_2O_2$
Exact Mass: 189.9588

Isolated yield: 0.171 g (69%, 1.30 mmol scale), white crystals (recrystallised from MeCN); $^1$H NMR (400 MHz, DMSO-$d_6$) δ/ppm 13.56 (s, br, 1H), 7.83 (d, $J$ = 8.4 Hz, 1H), 7.74 (d, $J$ = 2.1 Hz, 1H), 7.53 (dd, $J$ = 8.4, 2.1 Hz, 1H); $^{13}$C NMR (151 MHz, DMSO- $d_6$) δ/ppm 165.8 (C), 136.5 (C), 133.0 (C), 132.4 (CH), 130.2(CH), 130.1 (C), 127.5 (CH); IR (neat) ν = 1691 (s, C=O) cm$^{-1}$; LC-MS (MeCN), Rt. 2.85 min, m/z = 189.0 [M-H]$^-$. HR-MS ($^-$ESI-TOF) calculated for $C_7H_3O_2Cl_2$ 188.9510, found 188.9512 (Δ = 1.1 ppm). M.p. 162-163 °C (MeCN) (Literature: 162 °C).[13]

**2,5-dichlorobenzoic acid, [CAS Number: 50-79-3], 21:**

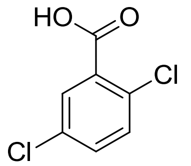


Chemical Formula: $C_7H_4Cl_2O_2$
Exact Mass: 189.9588

Isolated yield: 0.118 g (48%, 1.30 mmol scale), white crystals (recrystallised from MeCN); $^1$H NMR (400 MHz, DMSO-$d_6$) δ/ppm 13.73 (s, br, 1H), 7.82 (d, $J$ = 2.4 Hz, 1H), 7.66 – 7.54

(m, 2H); $^{13}$C NMR (151 MHz, DMSO- $d_6$) δ/ppm 165.5 (C), 133.2 (C), 132.34 (CH), 132.25 (CH), 131.8 (C), 130.3 (C), 130.2 (CH); IR (neat) ν = 1675 (s, C=O) cm$^{-1}$; LC-MS (MeCN), Rt. 2.52 min, m/z = 189.0 $[M-H]^-$. HR-MS ($^-$ESI-TOF) calculated for $C_7H_3O_2Cl_2$ 188.9510, found 188.9518 (Δ = 4.2 ppm). M.p. 153-155 °C (MeCN) (Literature: 155-156 °C).[14]

**2-chloro-5-(trifluoromethyl)benzoic acid, [CAS Number: 657-06-7], 22:**

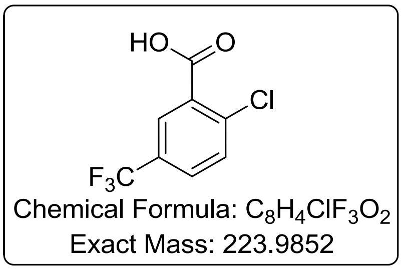


Isolated yield: 0.136 g (47%, 1.30 mmol scale), white crystals (recrystallised from $CHCl_3$); $^1$H NMR (400 MHz, DMSO-$d_6$) δ/ppm 13.92 (s, br, 1H), 8.09 (d, $J$ = 2.3 Hz, 1H), 7.91 (dd, $J$ = 8.5, 2.3 Hz, 1H), 7.83 – 7.78 (m, 1H); $^{13}$C NMR (101 MHz, DMSO- $d_6$) δ/ppm 166.0 (C), 136.4 (d, $J$ = 1.3 Hz, C), 133.2 (C), 132.4 (CH), 129.4 (q, $J$ = 3.4 Hz, CH), 128.3 (q, $J$ = 33.1 Hz, C), 127.9 (q, $J$ = 3.8 Hz, CH), 123.9 (q, $J$ = 273.5 Hz, C); $^{19}$F NMR (376 MHz, DMSO-$d_6$) δ/ppm -61.34 (s); IR (neat) ν = 1690 (s, C=O) cm$^{-1}$; LC-MS (MeCN), Rt. 2.99 min, m/z = 223.0 $[M-H]^-$. HR-MS ($^-$ESI-TOF) calculated for $C_8H_3O_2F_3Cl$ 222.9774, found 222.9766 (Δ = 3.6 ppm). M.p. 81-83 °C ($CHCl_3$) (Literature: 91-93 °C).[9]

**2-bromo-5-formylbenzoic acid, [CAS Number: 1289007-84-6], 23:**

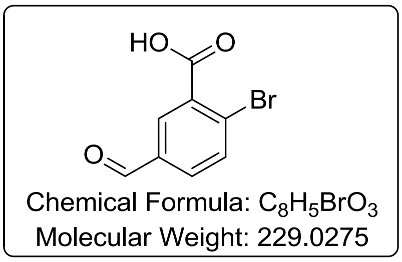


Isolated yield: 0.127 g (43%, 1.30 mmol scale), amorphous solid; $^1$H NMR (400 MHz, DMSO-$d_6$) δ/ppm 13.70 (s, br, 1H), 10.03 (s, 1H), 8.24 (d, $J$ = 2.1 Hz, 1H), 7.98 – 7.86 (m, 2H); $^{13}$C NMR (101 MHz, DMSO- $d_6$) δ/ppm 192.0 (CH), 166.6 (C), 135.2 (C), 135.0 (CH), 134.5 (C), 132.0 (CH), 131.5 (CH), 126.7 (C); IR (neat) ν = 1663 (s, C=O), 1731 (s, C=O) cm$^{-1}$; LC-MS (MeCN), Rt. 2.05 min, m/z = 227.0 $[M-H]^-$. HR-MS ($^-$ESI-TOF) calculated for $C_8H_4BrO_3$ 226.9344, found 226.9352 (Δ = 3.5 ppm).

**2-fluoro-5-formylbenzoic acid, [CAS Number: 550363-85-4], 24:**

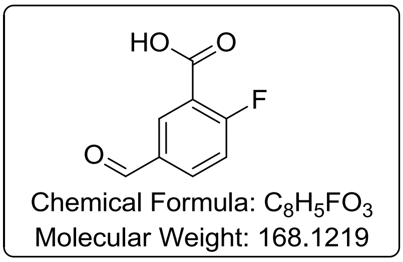


Isolated yield: 0.102 g (47%, 1.30 mmol scale), yellow crystals (recrystallised from MeCN); $^1$H NMR (400 MHz, DMSO-$d_6$) δ/ppm 13.66 (s, br, 1H), 10.04 (s, 1H), 8.43 (dd, $J$ = 7.2, 2.3

Hz, 1H), 8.17 (ddd, *J* = 8.5, 4.7, 2.3 Hz, 1H), 7.56 (dd, *J* = 10.6, 8.5 Hz, 1H); $^{13}$C NMR (151 MHz, DMSO- $d_6$) δ/ppm 191.8 (s, CH), 164.8 (d, *J* = 266.0 Hz, C), 164.5 (d, *J* = 3.1 Hz, C), 135.5 (d, *J* = 10.9 Hz, CH), 134.5 (d, *J* = 2.7 Hz, CH), 133.1 (d, *J* = 3.1 Hz, C), 120.6 (d, *J* = 11.3 Hz, C), 118.7 (d, *J* = 23.6 Hz, CH); $^{19}$F NMR (376 MHz, DMSO-$d_6$) δ/ppm -101.25 (s); IR (neat) ν = 1701 (s, C=O), 1685 (s, C=O) cm$^{-1}$; LC-MS (MeCN), Rt. 1.93 min, m/z = 167.1 [M-H]$^-$. HR-MS ($^-$ESI-TOF) calculated for $C_8H_4FO_3$ 167.0144, found 167.0146 (Δ = 1.2 ppm). M.p. 162 °C (MeCN, decomposed).

**2-Bromo-5-(ethoxymethyl)benzoic acid, 25:**

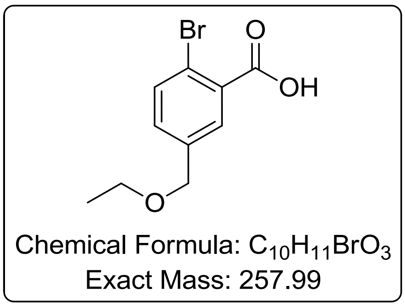


Isolated yield: 0.156 g (36%, 1.00 mmol scale), amorphous solid; $^1$H NMR (400 MHz, $CDCl_3$) δ/ppm 10.40 (s, br, 1H), 7.96 (d, *J* = 2.2 Hz, 1H), 7.67 (d, *J* = 8.3 Hz, 1H), 7.38 (dd, *J* = 8.2, 2.2 Hz, 1H), 4.51 (s, 2H), 3.57 (q, *J* = 7.0 Hz, 2H), 1.26 (t, *J* = 7.1, 3H); $^{13}$C NMR (101 MHz, $CDCl_3$) δ/ppm 170.9 (C), 138.4 (C), 135.0 (CH), 132.7 (CH), 131.5 (CH), 130.5 (C), 121.5 (C), 71.4 ($CH_2$), 66.3 ($CH_2$), 15.3 ($CH_3$); IR (neat) ν = 1699 (s, C=O) cm$^{-1}$; LC-MS (MeCN), Rt. 2.15 min, m/z = 259.3 [M+H]$^+$. HR-MS ($^+$ESI-TOF) calculated for $C_{10}H_{12}BrO_3$ 258.9970, found 258.9965 (Δ = 1.9 ppm).

**(*rac*)-(2-bromo-5-((2*R*,3*S*,5*S*)-3-(*tert*-butoxycarbonyl)-5-(ethoxycarbonyl)-3-methyl-1-pivaloylpyrrolidin-2-yl)benzoic acid, 26:**

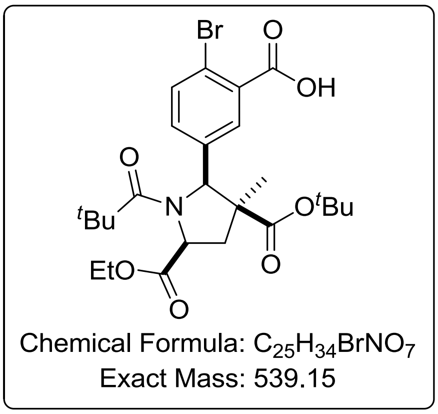


Isolated yield: 0.366 g (54%, 1.76 mmol scale), colourless crystals (recrystallised from hexane/EtOAc); $^1$H NMR (700 MHz, $CDCl_3$) δ/ppm 10.02 (s, br, 1H), 8.14 (s, 1H), 8.11 – 8.00 (m, 1H), 7.69 (d, *J* = 8.4 Hz, 1H), 5.09 (s, 1H), 4.55 (dd, *J* = 12.0, 7.1 Hz, 1H), 4.29 (qd, *J* = 7.1, 1.7 Hz, 2H), 2.49 (t, *J* = 12.7 Hz, 1H), 1.99 (dd, *J* = 13.2, 7.1 Hz, 1H), 1.46 (s, 3H), 1.33 (t, *J* = 7.2 Hz, 3H), 1.17 (s, 9H), 1.07 (s, 9H). $^{13}$C NMR (176 MHz, $CDCl_3$) δ/ppm 178.9 (C), 172.4 (C), 170.8 (C), 170.2 (C), 139.8 (C), 135.1 (CH), 133.5 (CH), 132.7 (CH), 130.1 (C), 122.0 (C), 82.3 (C), 69.7 (CH), 61.5 ($CH_2$), 61.2 (CH), 56.3 (C), 39.7 (C), 32.8 ($CH_2$), 28.4 ($CH_3$), 27.7 ($CH_3$), 23.7 ($CH_3$), 14.3 ($CH_3$); IR (neat) ν = 1723 (s, C=O), 1627 (m, amide I band) cm$^{-1}$; LC-MS (MeCN), Rt. 3.30 min, m/z = 540.5 [M+H]$^+$. HR-MS ($^+$ESI-TOF)

calculated for $C_{25}H_{24}BrNO_7$ 540.1597, found 540.1582 (Δ = 2.8 ppm), M.p. 162-165 °C (hexane/EtOAc).

**2,6-dimethoxybenzoic acid, [CAS Number: 1466-76-8], 27:**

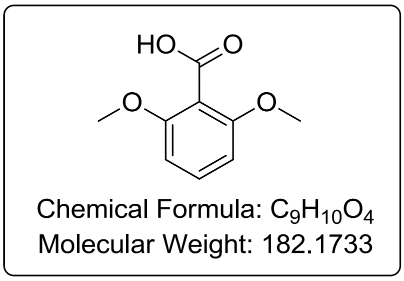


Isolated yield: 0.042 g (16%, 1.30 mmol scale), white crystals (recrystallised from MeCN); $^{1}H$ NMR (400 MHz, DMSO-*d*$_6$) δ/ppm 12.72 (s, br, 1H), 7.32 (t, *J* = 8.4 Hz, 1H), 6.69 (d, *J* = 8.4 Hz, 2H), 3.76 (s, 6H); $^{13}C$ NMR (151 MHz, DMSO- *d*$_6$) δ/ppm 167.2 (C), 156.6 (C), 130.9 (CH), 114.8 (C), 104.6 (CH), 56.2 ($CH_3$); IR (neat) ν = 1695 (s, C=O) $cm^{-1}$; LC-MS (MeCN), Rt. 2.07 min, m/z = 181.1 $[M-H]^-$. HR-MS ($^-$ESI-TOF) calculated for $C_9H_9O_4$ 181.0501, found 181.0508 (Δ = 3.9 ppm). M.p. 184-187 °C (MeCN) (Literature: 185-187 °C).[15]

**(4-Bromo-3-iodophenyl)methanol, 30:**

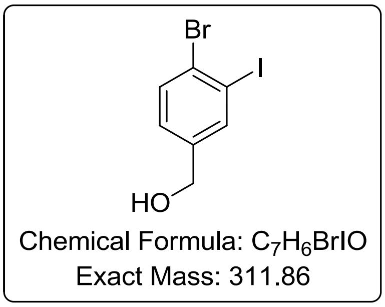


To a suspension of 4-bromo-3-iodobenzaldehyde (1.24 g, 4.0 mmol) in methanol (6 mL) at 0 ºC, $NaBH_4$ (0.08 g, 2.0 mmol) was added in small portions over 10 minutes. The reaction mixture was allowed to warm to room temperature and left stirring for 1 h. The solvent of the reaction mixture was then evaporated under vacuum and the residue was dissolved in ethyl acetate (25 mL) and washed with brine solution (3 x 25 mL). The organic layer was dried over sodium sulphate and the solvent evaporated under vacuum to give the desired product as white solid which was used without further purification.

Isolated yield: 1.17 g (93%, 4.00 mmol scale), white crystals (recrystallised from $CHCl_3$); $^{1}H$ NMR (400 MHz, $CDCl_3$) δ/ppm δ 7.85 – 7.81 (d, *J* = 4.0 Hz, 1H), 7.55 (d, *J* = 8.2 Hz, 1H), 7.17 – 7.12 (dd, *J* = 4.0, 8.0 Hz 1H), 4.56 (s, 2H), 2.35 (s, br, 1H); $^{13}C$ NMR (101 MHz, $CDCl_3$) δ/ppm 141.5 (C), 138.6 (CH), 132.7 (CH), 128.7 (C), 128.0 (CH), 101.4 (C), 63.3 ($CH_2$); IR (neat) ν = 3293 (br, C-OH), 2921 (w), 1450 (m), 1387 (m), 1195 (w), 1101 (m), 1006 (s), 808 (s) $cm^{-1}$; LC-MS (MeCN), Rt. 2.14 min, m/z = 295.14 $[M-H_2O]^+$. HR-MS ($^+$ESI-TOF) calculated for $C_7H_5BrI$ 294.8619, found 294.8625 (Δ = 2.0 ppm). M.p. 44-47 °C ($CHCl_3$).

**1-Bromo-4-(ethoxymethyl)-2-iodobenzene, 31:**

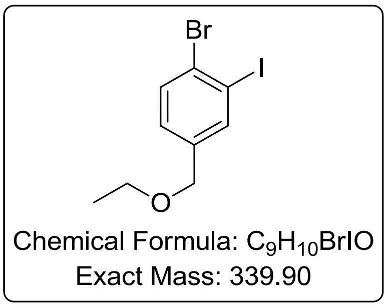


To a solution of (4-bromo-3-iodophenyl)methanol (**30**) (1.15 g, 3.67 mmol) in THF (4 mL), NaH (60% in hexane, 0.224 g, 5.5 mmol) was added in small portions over 5 minutes while keeping the reaction mixture at 0 ºC. Iodoethane (0.860 g, 0.44 mL, 5.5 mmol) was then added to the reaction mixture which was then allowed to warm up to room temperature and left stirring for 2 h. The solvent of the reaction mixture was then evaporated under vacuum and the residue was dissolved in ethyl acetate (25 mL) and washed with brine solution (3 x 25 mL). The organic layer was dried over sodium sulphate and the solvent evaporated under vacuum to give the desired product as yellow oil which was purified using flash silica chromatography 0.5:9.5 – 2:3 EtOAc/hexane gradient to give the product as a colourless liquid.

Isolated yield: 0.698 g (56%, 3.67 mmol scale), colourless liquid, $R_f$: 0.32 (2/8, EtOAc/hexane); $^{1}$H NMR (400 MHz, $CDCl_3$) δ/ppm 7.87 (dt, *J* = 1.9, 0.7 Hz, 1H), 7.60 (d, *J* = 8.2 Hz, 1H), 7.20 (ddt, *J* = 8.2, 2.0, 0.7 Hz, 1H), 4.43 (d, *J* = 0.8 Hz, 2H), 3.56 (q, *J* = 7.0 Hz, 2H), 1.27 (t, *J* = 7.0 Hz, 3H); $^{13}$C NMR (101 MHz, $CDCl_3$) δ/ppm 139.5 (C), 139.2 (CH), 132.5 (CH), 128.6 (CH), 128.5 (C), 101.1 (C), 71.0 ($CH_2$), 66.1 ($CH_2$), 15.2 ($CH_3$); IR (neat) ν = 2974 (w), 2865 (w), 1454 (m), 1383 (m), 1100 (s), 1009 (m), 811 (m) $cm^{-1}$; GC-MS (MeCN), Rt. 4.72 min, m/z = 340.0 $[M]^+$. LC-MS (MeCN), Rt. 4.31 min, m/z = 294.86 $[M\text{-}EtOH]^+$, HR-MS ($^+$ESI-TOF) calculated for $C_7H_5BrI$ 294.8619, found 294.8611 (Δ = 2.7 ppm), ASAP (MeCN), Rt. 0.51 min, m/z = 335.9 $[M+EtOH+MeCN]^+$. HR-MS ($^+$AP-TOF) calculated for $C_9H_8BrIN$ 335.8885, found 335.8875 (Δ = 3.0 ppm), ASAP (MeCN), Rt. 0.51 min, m/z = 381.9 $[M+H+MeCN]^+$. HR-MS ($^+$AP-TOF) calculated for $C_{11}H_{14}BrINO$ 381.9304, found 335.9297 (Δ = 1.8 ppm).

**(*rac*)-(2*S*,4*S*,5*R*)-4-*tert*-butyl 2-ethyl 5-(4-bromo-3-iodophenyl)-4-methylpyrrolidine-2,4-dicarboxylate, 32:**

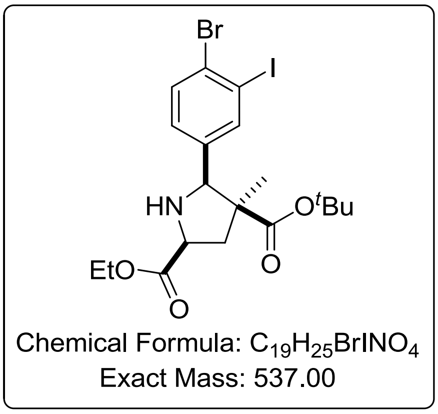


To a suspension of 4-bromo-3-iodobenzaldehyde (1.24 g, 4.0 mmol) and glycine hydrochloride (0.838 g, 6.0 mmol) in acetonitrile (6 mL), triethylamine (0.836 g, 6.0 mmol)

was added and reaction mixture was left stirring at room temperature for 4 h. The solvent of the reaction mixture was then evaporated under vacuum and the residue was dissolved in ethyl acetate (25 mL) and washed with brine solution (3 x 25 mL). The organic layer was dried over sodium sulphate and the solvent evaporated under vacuum to give the desired imine intermediate which was used in the next step without further purification. The imine intermediate was dissolved in THF (6 mL) and *tert*-butyl methacrylate (1.138 g, 8.0 mmol) was added followed by lithium bromide (0.694 g, 8.0 mmol) and triethylamine (1.11 g, 8.0 mmol). The reaction mixture was left stirring at room temperature for 2 h after which the solvent of the reaction mixture was then evaporated under vacuum and the residue was dissolved in ethyl acetate (25 mL) and washed with brine solution (3 x 25 mL). The organic layer was dried over sodium sulphate and the solvent evaporated under vacuum to give the desired crude product as a yellow oil which was purified using flash silica chromatography 1:9 EtOAc/hexane.

Isolated yield: 2.00 g (93%, 4.0 mmol scale), yellow oil, $R_f$: 0.15 (2/8, EtOAc/hexane); $^1$H NMR (600 MHz, $CDCl_3$) δ/ppm; 7.83 (d, *J* = 2.1 Hz, 1H), 7.52 (d, *J* = 8.3 Hz, 1H), 7.22 (dd, *J* = 8.3, 2.2 Hz, 1H), 4.25 (q, *J* = 7.1 Hz, 2H), 3.98 – 3.92 (m, 2H), 2.73 (s, br, 1H), 2.60 (dd, *J* = 13.2, 8.9 Hz, 1H), 2.04 (dd, *J* = 13.2, 8.1 Hz, 1H), 1.42 (s, 3H), 1.31 (t, *J* = 7.1 Hz, 3H), 1.12 (s, 9H). $^{13}$C NMR (151 MHz, $CDCl_3$) δ/ppm 173.6 (C), 173.0 (C), 141.5 (C), 139.3 (CH), 132.2 (CH), 128.6 (CH), 128.5 (C), 100.9 (C), 81.2 (C), 71.4 (CH), 61.3 ($CH_2$), 58.8 (CH), 55.1 (C), 41.3 ($CH_2$), 27.7 ($CH_3$), 24.4 ($CH_3$), 14.4 ($CH_3$); IR (neat) ν = 2997 (w), 1933 (w), 1717 (s, C=O), 1449 (m), 1367 (m), 1248 (s), 1149 (s), 1110 (s), 1033 (m), 1009 (m), 847 (m) $cm^{-1}$; LC-MS (MeCN), Rt. 3.77 min, m/z = 538.4 $[M+H]^+$. HR-MS ($^+$ESI-TOF) calculated for $C_{19}H_{25}BrINO_4$ 538.0090, found 538.0073 (Δ = 3.2 ppm).

**(*rac*)-(2*S*,4*S*,5*R*)-4-*tert*-butyl 2-ethyl 5-(4-bromo-3-iodophenyl)-1-ethyl-4-methylpyrrolidine-2,4-dicarboxylate, 29:**

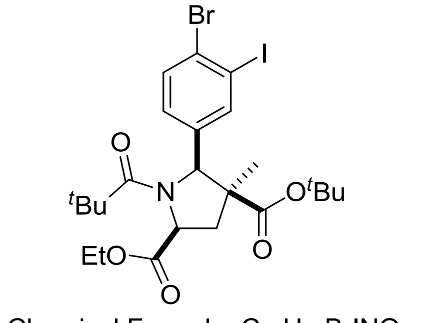

Chemical Formula: $C_{24}H_{33}BrINO_5$
Exact Mass: 621.06

To a solution **32** (2.0 g, 3.72 mmol) in dichloromethane (4 mL), pivolyl chloride (0.580 g, 0.594 mL, 4.83 mmol) was added followed by triethylamine (0.489 g, 0.674 mL, 4.83 mmol) and reaction was left stirring at room temperature for 2 h. The reaction mixture was then washed with brine solution (3 x 25 mL) and the organic layer dried over sodium sulphate and the solvent evaporated under vacuum to give the desired product as a yellow oil which was purified using flash silica chromatography 1:9 EtOAc/hexane.

Isolated yield: 1.49 g (65%, 3.72 mmol scale), white crystals (recrystallised from $CH_3Cl$), $R_f$: 0.24 (2/8, EtOAc/hexane); $^1$H NMR (600 MHz, $CDCl_3$) δ/ppm 8.04 (s, 1H), 7.83 (d, *J* = 8.3 Hz, 1H), 7.57 (d, *J* = 8.3 Hz, 1H), 4.95 (s, 1H), 4.50 (dd, *J* = 12.0, 7.1 Hz, 1H), 4.29 (q, *J* = 7.1 Hz, 2H), 2.46 (t, *J* = 12.6 Hz, 1H), 1.96 (dd, *J* = 13.1, 7.1 Hz, 1H), 1.42 (s, 3H), 1.33 (t, *J* = 7.1 Hz, 3H), 1.22 (s, 9H), 1.07 (s, 9H); $^{13}$C NMR (151 MHz, $CDCl_3$) δ/ppm δ 178.5 (C),

172.2 (C), 170.5 (C), 140.8 (C), 140.1 (CH), 132.4 (CH), 129.5 (CH), 129.0 (C), 100.7 (C), 82.1 (C), 69.0 (CH), 61.2 ($CH_2$), 60.9 (CH), 56.1 (C), 39.5 (C), 32.6 ($CH_2$), 28.3 ($CH_3$), 27.7 ($CH_3$), 23.5 ($CH_3$), 14.2 ($CH_3$); IR (neat) ν = 2975 (w), 2935 (w), 1743 (s, C=O), 1722 (s, C=O), 1632 (s, amide I band), 1456 (m), 1394 (m), 1251 (m), 1197 (s), 1167 (s), 1129 (s), 730 (s) $cm^{-1}$; LC-MS (MeCN), Rt. 4.05 min, m/z = 622.5 $[M+H]^+$. HR-MS ($^+$ESI-TOF) calculated for $C_{21}H_{29}BrINO_4$ 622.0665, found 622.0662 (Δ = 0.5 ppm).

*Crystal data*: **29**, $C_{24}H_{33}BrINO_5$, $M$=622.32, $T$=120 K, triclinic, space group $P\bar{1}$ (No. 2), $a$=8.8223(5), $b$=9.7894(5), $c$= 17.1881(9) Å, α= 94.479(2), β= 101.255(2), γ= 116.252(2)º, $V$= 1282.7(1) $Å^3$, $Z$=2, $D_c$=1.611 g $cm^{-3}$, μ=0.74 $mm^{-1}$, 34192 reflections with 2θ≤71.7º, 10848 unique, $R_{int}$=0.031, $R(F)$=0.027 [9096 data with $I$≥2σ($I$)], w$R$($F^2$)=0.059 (all data). CCDC-1470506.

References


1. Gottlieb, H. E.; Kotlyar, V.; Nudelman, A., *J. Org. Chem.* **1997**, *62* (21), 7512-7515.
2. Sheldrick, G., *Acta Crystallographica Section A* **2008**, *64* (1), 112-122.
3. Dolomanov, O. V.; Bourhis, L. J.; Gildea, R. J.; Howard, J. A. K.; Puschmann, H., *J. Appl. Crystallogr.* **2009**, *42* (2), 339-341.
4. Sheldrick, G., *Acta Crystallographica Section A* **2015**, *71* (1), 3-8.
5. Shaikh, T. M.; Hong, F.-E., *Tetrahedron* **2013**, *69* (42), 8929-8935.
6. Durka, K.; Lulinski, S.; Dabrowski, M.; Serwatowski, J., *Eur. J. Org. Chem.* **2014**, (21), 4562-4570.
7. Yonemoto-Kobayashi, M.; Inamoto, K.; Kondo, Y., *Chem. Lett.* **2014**, *43* (4), 477-479.
8. Sathyanarayana, P.; Ravi, O.; Muktapuram, P. R.; Bathula, S. R., *Org. Biomol. Chem.* **2015**, *13* (37), 9681-9685.
9. Mongin, F.; Desponds, O.; Schlosser, M., *Tetrahedron Lett.* **1996**, *37* (16), 2767-2770.
10. Chi-Chung Choy, J.; Jaime-Figueroa, S. Deprotection of BOC-Protected Compounds. US 2010/0311968 A1, 2010.
11. Thirumamagal, B. T. S.; Narayanasamy, S.; Venkatesan, R., *Synth. Commun.* **2008**, *38* (16), 2820-2825.
12. Sturm, K.; Siedel, W.; Weyer, R.; Ruschig, H., *Chem. Ber. Recl.* **1966**, *99* (1), 328-&.
13. Ley, C. P.; Yates, M. H., *Org. Process. Res. Dev.* **2008**, *12* (1), 120-124.
14. Tong, L. K. J.; Kenyon, W. O., *J. Am. Chem. Soc.* **1947**, *69* (6), 1402-1405.
15. Phillion, D. P.; Pratt, J. K., *Synth. Commun.* **1992**, *22* (1), 13-22.
